# Supplementary material for: Metapopulation dynamics of SARS-CoV-2 transmission in a small-scale Amazonian society
Source: PLoS Biol. 2023 Aug 22;21(8):e3002108. doi: 10.1371/journal.pbio.3002108 (PMC10443873; doi:10.1371/journal.pbio.3002108)
Supplement: S1 Text — (DOCX) [file pbio.3002108.s002.docx]

**Supporting text for “Metapopulation dynamics of SARS-CoV-2 transmission in a small-scale Amazonian society”:**

**Supplementary Materials and Methods**

***Empirical data on micro-level processes***

*Kinship:* Genealogical records were used to construct matrices (7269 x 7269) representing biological relatedness (coefficient of relatedness, *r*, between individuals on rows and columns) and “affinal relatedness” (defined here as the coefficient of relatedness for an individual equal to that between one’s spouse and other individuals; e.g. affinal relatedness to spouse = 1, brother-in-law = 0.5).

*Spatial information:* As noted in the main text, GPS data were available for a large but incomplete proportion of our study sample. Missing data were imputed for use in our epidemiological model. If GPS data were available for >65% of individuals in a village, we used the kernel density of the focal community for imputation. If coverage was <65%, we first matched focal communities with other communities that had greater GPS coverage (<35% missing) of similar size (<15% difference in number of residents), and performed imputations at the household level centered on the coordinates of the focal community.

*Intervillage travel:* Because we lacked high quality information on visitation rates between all 65 focal communities, we were unable to estimate rates of between-village travel directly. Instead, we re-analyzed a previously published database of interviews in which Tsimane participants were asked about all of the locations they had visited outside of their home community in the past month, the frequency of visits to each location, and the reason for their visits [1]. The data were filtered to only include rows in which individuals traveled from their home community to another Tsimane community (i.e., excluding trips to Spanish towns). Using these data, we ran a binomial generalized linear model where each row represented a single individual with 30 overall trials (number of days in a month) and a given number of “successes” corresponding to the cumulative number of days spent outside of the home community. Predictor variables in this model included individual sex, age, and age^2^. This model was then used to calculate the expected daily probability of visiting another Tsimane community for individuals of different ages and sexes.

Next, we expanded the dataset so that for each day of reported travel for an individual, rows representing all Tsimane villages were generated. All rows received an outcome of 0 (did not travel there) for a given day except for the one location representing the village which was visited (assigned an outcome of 1). Columns representing attributes of villages were populated with information on distance from the focal individual’s home community (measured as expected travel time using the most common form of transportation; travel times between communities were estimated by interviewing locals about the time it takes to move between different areas, corroborating those values with locations on a map). We then fit a conditional logit model to these data using the *mlogit* package in R to characterize the effect of distance on the choice of community to visit, conditional upon having left one’s home community. Combining this with the predictions of the model described above, we were thus able to calculate the expected daily probability of visiting each other Tsimane village in the landscape based on individual age, sex, and home community location, resulting in a unique vector of travel probabilities for each individual in the model population.

In addition, one focal Tsimane community was expected to exhibit higher visitation rates due to its geographic proximity to town and its status as a central river port. Longitudinal interview data from this location estimated the number of boats arriving from other communities each day, the number of individuals in those boats, and their place of origin. We used these data to estimate the additional probability of travel to this community from other communities by calculating per capita rates based on the size of origin communities, and added the additional probability of travel to the port community from each alter community.

***Tie dissolution***

In most uses of *EpiModel* and individual network modeling with TERGM, a user will specify a baseline tie dissolution rate (the inverse of the expected average duration of a tie) and/or independent variables governing a heterogeneous dissolution model. This is especially useful when using egocentric network data where the duration of ties can be empirically parameterized (i.e. HIV networks in which transmission dynamics depend critically on the duration of sexual partnerships [2]). One limitation of our empirical data is that we lack a quantitative estimate of the distribution of tie durations to parameterize the rate of tie dissolution. The underlying empirical data do, however, provide a detailed description of the heterogeneity and preferential formation of ties on any given day. As such, we set the average tie duration to 1 day such that ties are resimulated for each time step of the model as a panel of cross-sectional ERGMs. Under this scenario, ties will persist across time steps (duration > 1) when the dyadic probability of formation is sufficiently high, such as in cases of close kinship or shared household. We set this parameter to reflect the fact that ties among Tsimane are relatively fluid over time, but within the confines of target statistics that promote homophily (e.g. within households, between related individuals, etc.). A dissolution duration of 1 day also accommodates a metapopulation system with frequent migration which necessitates the manual dissolution of ties when an individual leaves her resident community (which would require dynamic modification of formation/dissolution rates in a TERGM framework). Our code is customizable to employ longer tie durations or heterogeneous dissolution parameters as desired.

***Transmissibility parameter***

Some model parameters remain poorly known. In particular, transmissibility, which represents the probability that a susceptible node becomes infected while sharing a tie with an infected node for one timestep in our model, cannot be easily established for SARS-CoV-2 in an outdoor-living population in the Bolivian Amazon where there is a lack of sanitation facilities and clean water, and food, beds, and tools are frequently shared. Studies in other contexts have identified tremendous variability. A serological assay study in Norway estimated a household attack rate (probability of a susceptible individual in a shared household becoming infected following an index case) of 45% (95% CI: [38, 53]) [3] versus 17.1% [13.3, 21.8] in Guangzhou, China [4], and a meta-analysis across countries identified rates between 4.6% and 49.6% [5]. More directly, estimates of the secondary attack rate (SAR; probability that a susceptible individual becomes infected within a reasonable incubation period after contact with an infected individual in household or another close-contact environment) range between 0.5% and 63.9% [6–9]. In perhaps the most relevant environmental comparisons, Karumanagoundar et al. [10] and Ge et al. [11] reported overall SARs of 4% and 3.6% in studies of contacts in Tamil Nadu, India and Zhejiang, China, respectively. In contrast, a recent meta-analysis yielded median pooled SAR of 0.9% using data from 15 case series across a variety of settings in Taiwan (though this outcome likely reflects highly efficacious mitigation strategies and widespread use of facial masks) [12]. The attack rate may also vary by age and environmental setting. A study of two index cases in a church in Arkansas, for example, found that 38% of church participants were infected after attending events over a 5-day period, with the attack rate varying between 6.3% (ages <= 18) and 59.4% (ages 19-64) [13]. There also is evidence that the pathogenesis of SARS-CoV-2 varies in relation to altitude, humidity, and other environmental factors [14]. A better understanding of the factors affecting this crucial parameter is critical to forecasting COVID-19 impact across populations.

**References**

1. Miner EJ, Gurven MD, Kaplan H, Gaulin SJC. Sex difference in travel is concentrated in adolescence and tracks reproductive interests. Proceedings of the Royal Society B: Biological Sciences. 2014;281: 20141476.

2. Goodreau SM, Rosenberg ES, Jenness SM, Luisi N, Stansfield SE, Millett GA, et al. Sources of racial disparities in HIV prevalence in men who have sex with men in Atlanta, GA, USA: a modelling study. Lancet HIV. 2017;4: e311–e320. doi:10.1016/S2352-3018(17)30067-X

3. Kuwelker K, Zhou F, Blomberg B, Lartey S, Brokstad KA, Trieu MC, et al. Attack rates amongst household members of outpatients with confirmed COVID-19 in Bergen, Norway: a case-ascertained study. The Lancet Regional Health – Europe. 2021;3: 100014. doi:10.1016/J.LANEPE.2020.100014

4. Jing QL, Liu MJ, Zhang Z Bin, Fang LQ, Yuan J, Zhang AR, et al. Household secondary attack rate of COVID-19 and associated determinants in Guangzhou, China: a retrospective cohort study. Lancet Infect Dis. 2020;20: 1141–1150. doi:10.1016/S1473-3099(20)30471-0

5. Shah K, Saxena D, Mavalankar D. Secondary attack rate of COVID-19 in household contacts: a systematic review. QJM: An International Journal of Medicine. 2020;113: 841–850. doi:10.1093/QJMED/HCAA232

6. Liu Y, Eggo RM, Kucharski AJ. Secondary attack rate and superspreading events for SARS-CoV-2. The Lancet. 2020;395: e47. doi:10.1016/S0140-6736(20)30462-1

7. COVID-19 National Emergency Response Center. Coronavirus disease-19: summary of 2,370 contact investigations of the first 30 cases in the Republic of Korea. Osong Public Health and Research Perspectives. 2020;11: 81–84.

8. Burke R, Midgley C, Dratch A, Fenstersheib M, Haupt T, Holshue M, et al. Active monitoring of persons exposed to patients with confirmed COVID-19: United States, January-February 2020. Morbidity and Mortality Weekly Report. 2020;69: 245–246. Available: https://www.ncbi.nlm.nih.gov/pmc/articles/pmc7367094/

9. Sun W, Ling F, Pan J, Cai J, Miao Z, Liu S, et al. Epidemiological characteristics of COVID-19 family clustering in Zhejiang Province. Chinese Journal of Preventive Medicine. 2020;54: 625–629. doi:10.3760/CMA.J.CN112150-20200227-00199

10. Karumanagoundar K, Raju M, Ponnaiah M, Kaur P, Viswanathan V, Rubeshkumar P, et al. Secondary attack rate of COVID-19 among contacts and risk factors, Tamil Nadu, March–May 2020: a retrospective cohort study. BMJ Open. 2021;11: e051491. doi:10.1136/bmjopen-2021-051491

11. Ge Y, Martinez L, Sun S, Chen Z, Zhang F, Li F, et al. COVID-19 transmission dynamics among close contacts of index patients with COVID-19: a population-based cohort study in Zhejiang Province, China. JAMA Intern Med. 2021;181: 1343–1350. doi:10.1001/jamainternmed.2021.4686

12. Huang YT, Tu YK, Lai PC. Estimation of the secondary attack rate of COVID-19 using proportional meta-analysis of nationwide contact tracing data in Taiwan. Journal of Microbiology, Immunology and Infection. 2021;54: 89–92. doi:10.1016/J.JMII.2020.06.003

13. James A, Eagle L, Phillips C, Hedges DS, Bodenhamer C, Brown R, et al. High COVID-19 attack rate among attendees at events at a church — Arkansas, March 2020. Morbidity and Mortality Weekly Report. 2020;69: 632–635. doi:10.15585/MMWR.MM6920E2

14. Arias-Reyes C, Zubieta-DeUrioste N, Poma-Machicao L, Aliaga-Raduan F, Carvajal-Rodriguez F, Dutschmann M, et al. Does the pathogenesis of SARS-CoV-2 virus decrease at high-altitude? Respir Physiol Neurobiol. 2020;277: 103443. doi:10.1016/J.RESP.2020.103443
