# Supplementary material for: Metapopulation dynamics of SARS-CoV-2 transmission in a small-scale Amazonian society
Source: PLoS Biol. 2023 Aug 22;21(8):e3002108. doi: 10.1371/journal.pbio.3002108 (PMC10443873; doi:10.1371/journal.pbio.3002108)
Supplement: S1 Table — (DOCX) [file pbio.3002108.s003.docx]

| **Model term** | ***Β* (SE)** | **z value** | ***P*** |
| --- | --- | --- | --- |
| Edges (intercept) | 0.830 (0.038) | 21.9 | <0.001 |
| Sex (Male = 1) | -0.268 (0.014) | -19.2 | <0.001 |
| Sex homophily | 0.443 (0.020) | 21.7 | <0.001 |
| Age-mix (10-25 and 26-40) | -0.367 (0.030) | -12.4 | <0.001 |
| Age-mix (26-40 and 26-40) | -0.007 (0.045) | -0.2 | 0.87 |
| Age-mix (10-25 and 41-55) | -0.466 (0.037) | -12.7 | <0.001 |
| Age-mix (26-40 and 41-55) | 0.033 (0.044) | 0.8 | 0.45 |
| Age-mix (41-55 and 41-55) | 0.187 (0.073) | 2.6 | 0.01 |
| Age-mix (10-25 and 56-70) | 0.026 (0.044) | 0.6 | 0.56 |
| Age-mix (26-40 and 56-70) | 0.219 (0.060) | 3.7 | <0.001 |
| Age-mix (41-55 and 56-70) | 0.416 (0.076) | 5.5 | <0.001 |
| Age-mix (56-70 and 56-70) | -0.249 (0.198) | -1.3 | 0.21 |
| Age-mix (10-25 and 70+) | 0.172 (0.065) | 2.7 | 0.01 |
| Age-mix (26-40 and 70+) | 0.486 (0.084) | 5.8 | <0.001 |
| Age-mix (41-55 and 70+) | 0.618 (0.101) | 6.1 | <0.001 |
| Age-mix (56-70 and 70+) | 0.203 (0.190) | 1.1 | 0.28 |
| Age-mix (70+ and 70+) | -0.685 (0.423) | -1.6 | 0.11 |
| Genetic relatedness | 0.986 (0.069) | 14.2 | <0.001 |
| Affinal relatedness | 2.711 (0.076) | 35.5 | <0.001 |
| ln-Distance (km between households) | -0.799 (0.005) | -162.8 | <0.001 |
