# Supplementary figures and images for: Metapopulation dynamics of SARS-CoV-2 transmission in a small-scale Amazonian society
Source: PLoS Biol. 2023 Aug 22;21(8):e3002108. doi: 10.1371/journal.pbio.3002108 (PMC10443873; doi:10.1371/journal.pbio.3002108)

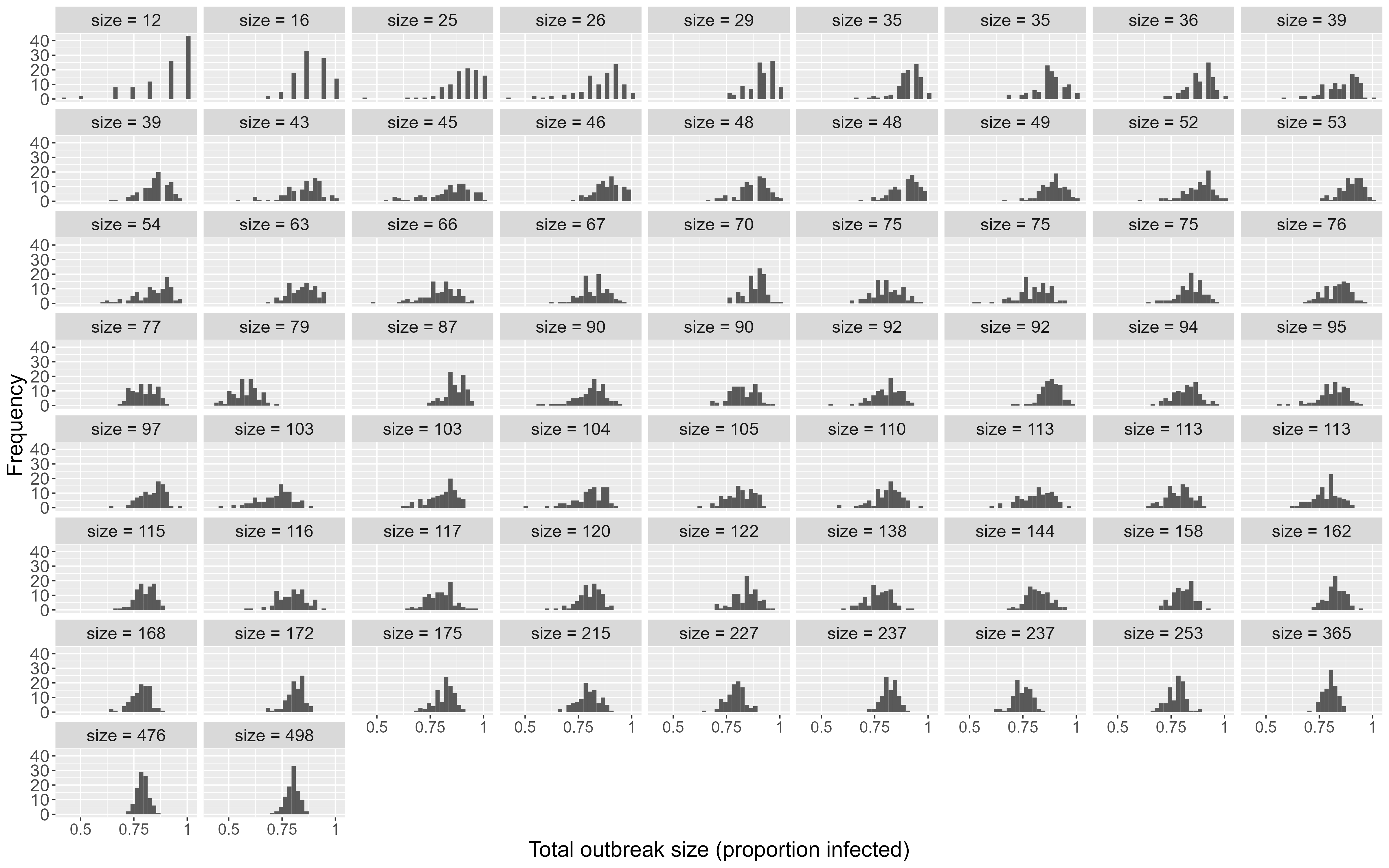

Supplement: S1 Fig — Communities are ordered by increasing size. Outcomes here are for simulations under baseline conditions. The data underlying this Figure can be found in http://doi.org/10.17605/OSF.IO/7YB2M (files: https://osf.io/jb5x7, https://osf.io/vtp3w). (TIFF) [file pbio.3002108.s004.tiff]

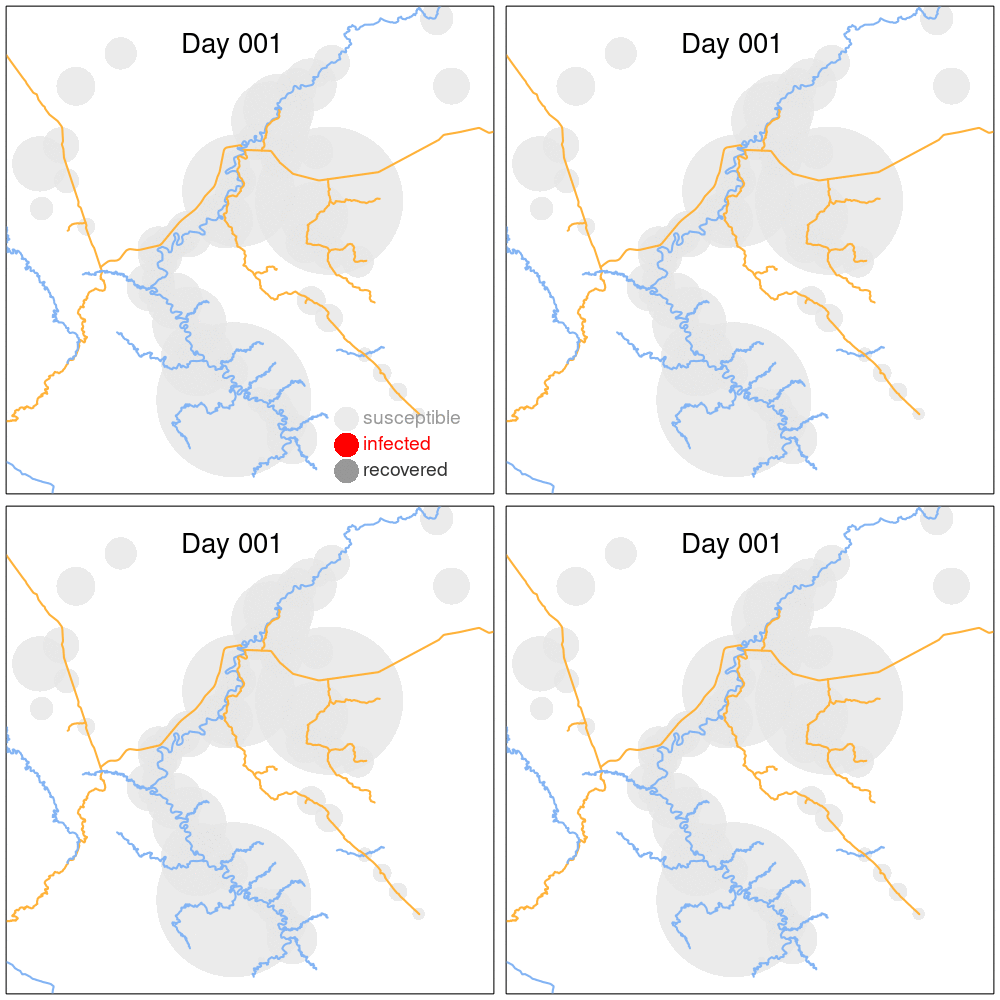

Supplement: S3 Fig — The fluctuating size of bubbles reflects movement in and out of resident communities (e.g., numbers represent individuals in a community at a given time point). https://thomaskraft.github.io/epidemic_simulations/landscape_spread_tsimane.gif. (GIF) [file pbio.3002108.s006.gif]

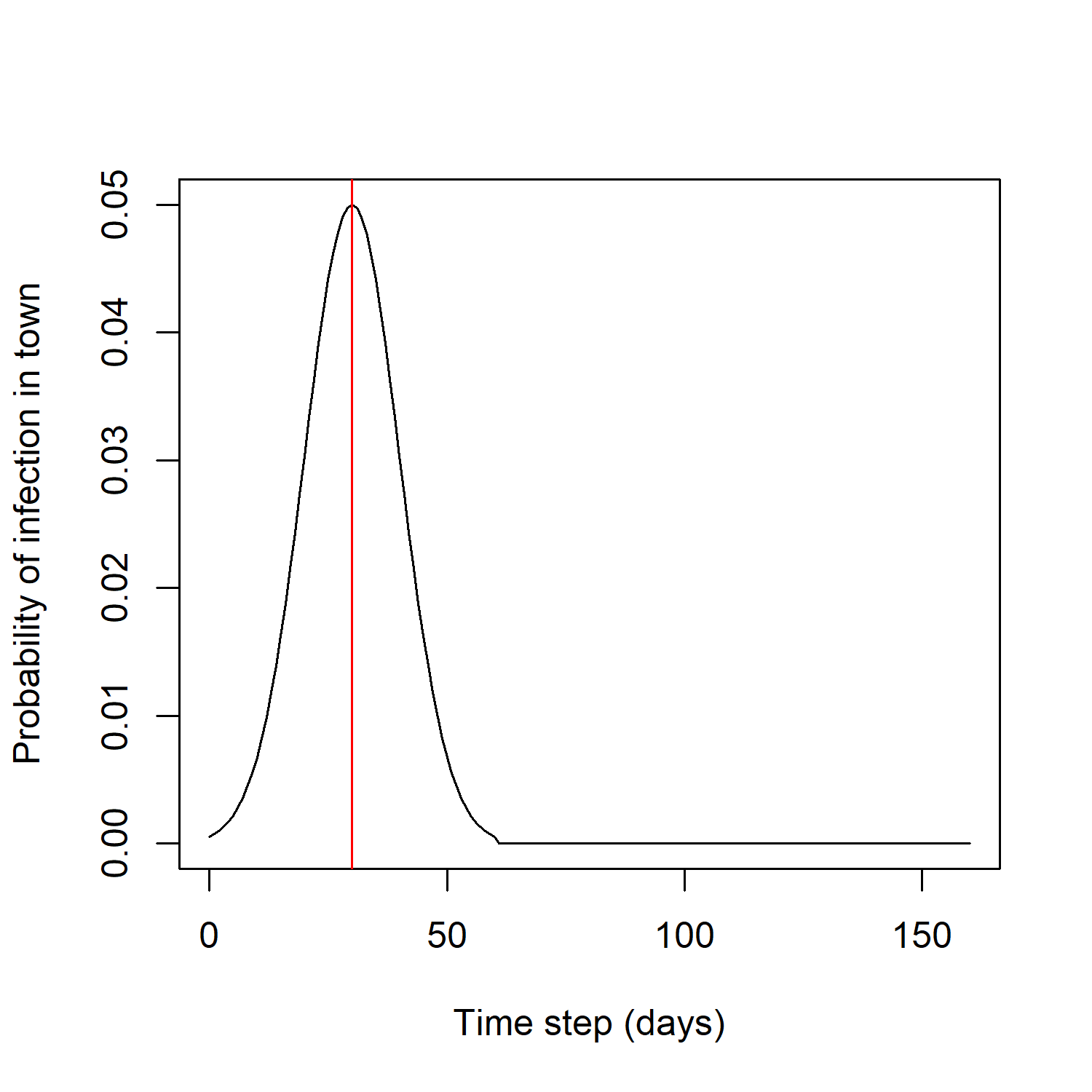

Supplement: S4 Fig — This profile assumes that it was possible to contract the disease in town over 2 months (60 days) from a single symmetric “wave,” peaking at a maximum 5% probability of contracting the disease (marked with red line). The data underlying this Figure can be found in http://doi.org/10.17605/OSF.IO/7YB2M (files: https://osf.io/m892d). (TIFF) [file pbio.3002108.s007.tiff]

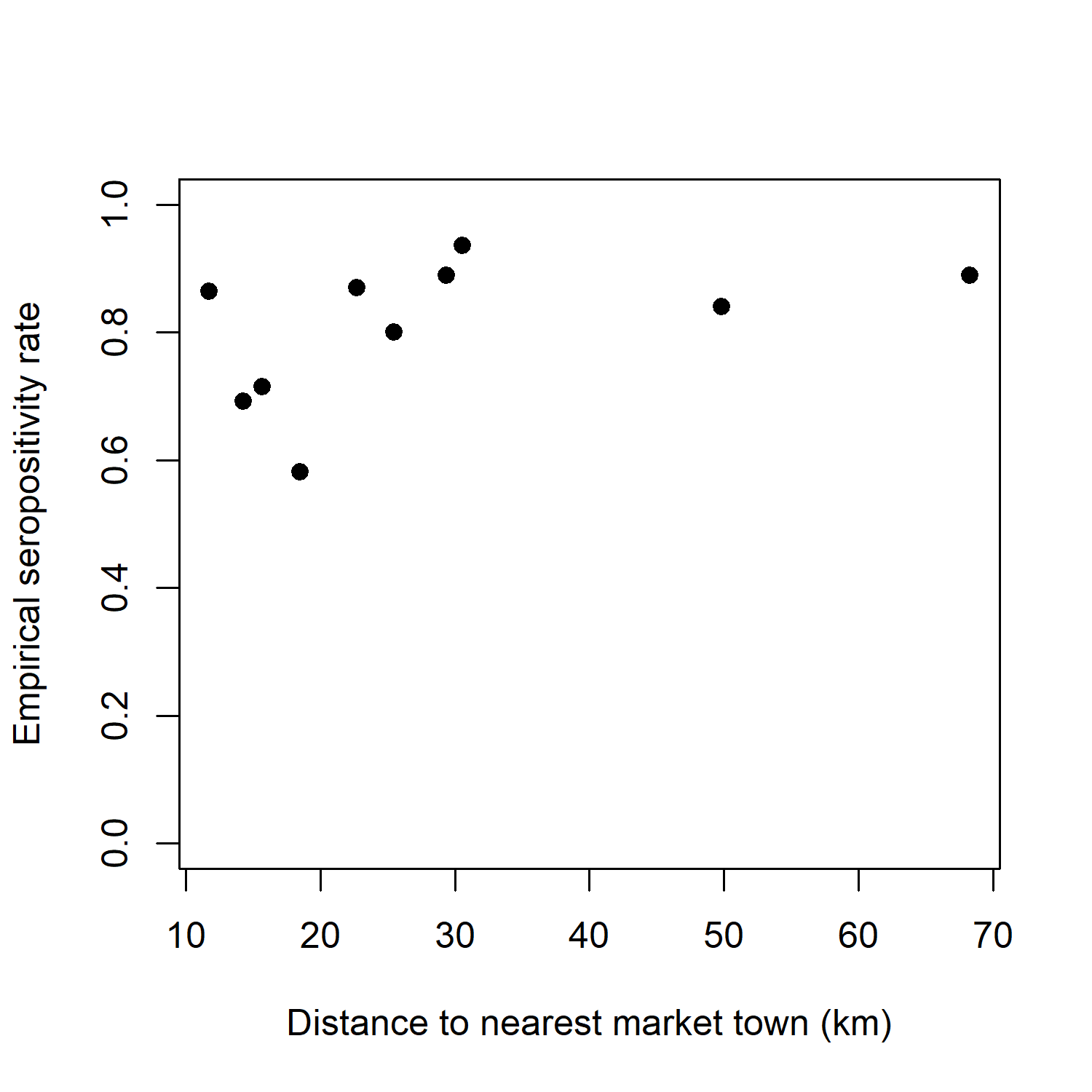

Supplement: S5 Fig — Each point represents a single community in which >20 seroassays were conducted. The data underlying this Figure can be found in http://doi.org/10.17605/OSF.IO/7YB2M (files: https://osf.io/3m7he, https://osf.io/jb5x7, https://osf.io/vtp3w). (TIFF) [file pbio.3002108.s008.tiff]

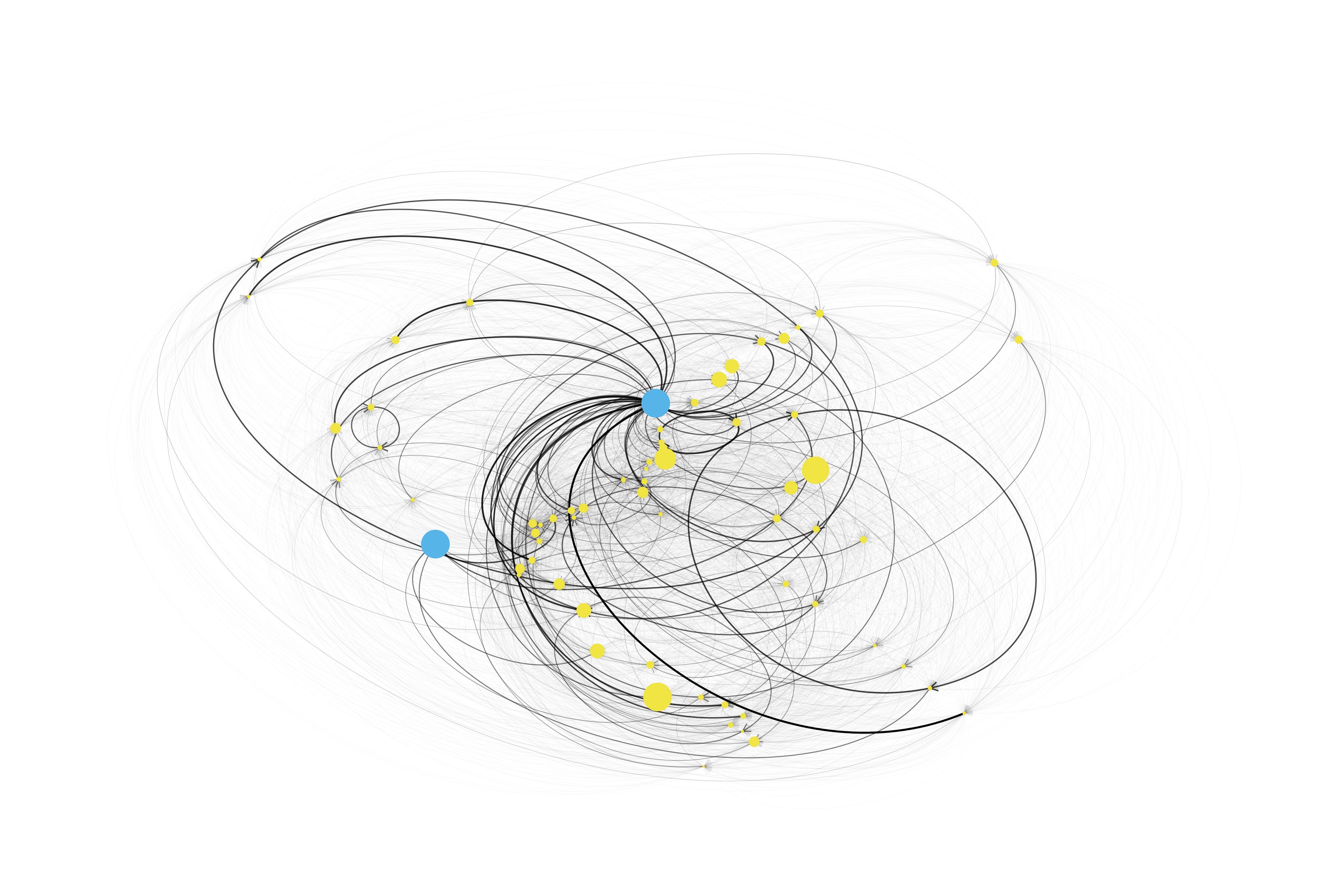

Supplement: S6 Fig — The thickness and transparency of edges is proportional to visitation probabilities in the baseline model. Yellow circles represent villages (size proportional to population size), and blue circles represent major market towns. Villages are displayed roughly according to location in geographic space. (PNG) [file pbio.3002108.s009.png]

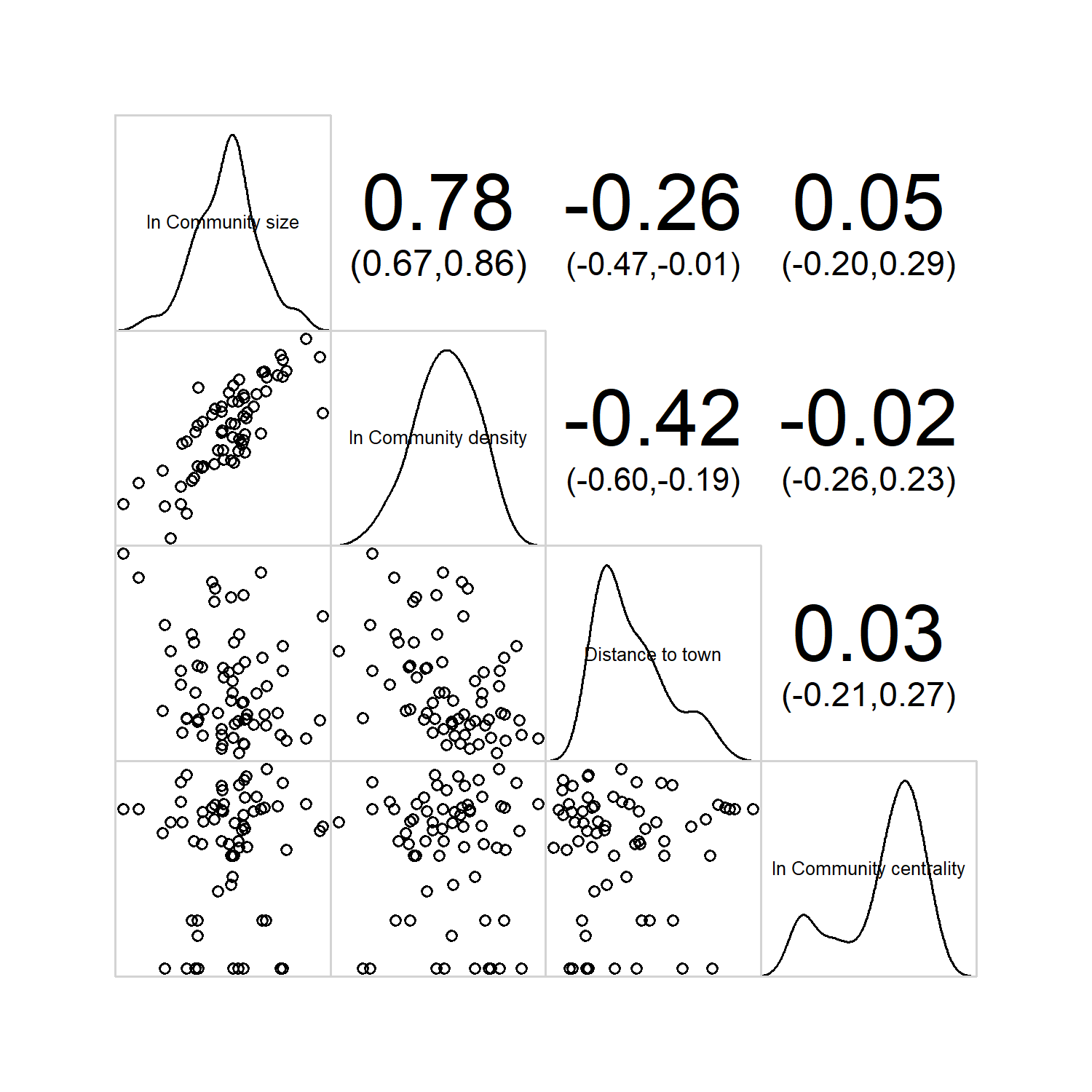

Supplement: S7 Fig — The upper triangle shows Pearson correlation coefficients (95% CIs), and the lower triangle shows bivariate scatterplots. Variance inflation factors for variables in community models are generally low with a maximum of 3.0 (community size–community density), indicating that multicollinearity is unlikely to be a problem for inference given large effect sizes and sample size. The data underlying this Figure can be found in http://doi.org/10.17605/OSF.IO/7YB2M (files: https://osf.io/vtp3w). (TIFF) [file pbio.3002108.s010.tiff]
