# Supplementary material for: Metapopulation dynamics of SARS-CoV-2 transmission in a small-scale Amazonian society
Source: PLoS Biol. 2023 Aug 22;21(8):e3002108. doi: 10.1371/journal.pbio.3002108 (PMC10443873; doi:10.1371/journal.pbio.3002108)
Supplement: Attachment — Submitted filename: Response to Reviewers.pdf [file pbio.3002108.s011.pdf]

Response to Reviewers

We appreciate the time and expert feedback provided by the editor, Dr. Roberts, and the two anonymous reviewers. Please see the point-by-point responses that follow each comment in bold for our responses.

Editor comments

a) Please could change your Title to "Metapopulation dynamics of SARS-CoV-2 transmission in a small-scale human Amazonian society"

This change has been made. The one exception is that we omitted the word "human" from the title because this seems reasonably implied.

b) Please read our "Inclusivity in Global Research" policy (https://journals.plos.org/plosbiology/s/best-practices-in-research-reporting#loc-inclusivity-in-global-research) and download, complete and upload the questionnaire.

This has been completed and added as a supplementary checklist. We note that this document asks for page numbers of some text, and we hope that this can be updated by an editor to reflect the page numbers of finalized typeset documents.

c) Please address the requests from reviewers #1 and #2.

Please see the point-by-point responses below.

d) Please attend to my Data Policy requests below; specifically, we need you to supply the numerical values underlying Figs 2AB, 3, 4ABCDEFGHIJKLMNOP, 5ABCDEF, 6ABCDEF, 7, S1, S4, S5, S6, S7, either as a supplementary data file or as a permanent DOI'd deposition. I note that you already have an associated GitHub deposition (https://github.com/ThomasKraft/tsimane_epidemic), but this URL does not appear to work; please rectify this. We're aware that the raw data cannot be shared because of privacy concerns, but please could you clarify whether the data/code are sufficient to reproduce your Figures? Also, because Github depositions can be readily changed or deleted, please make a permanent DOI'd copy (e.g. in Zenodo) and provide this URL (see below).

The numerical values requested for the listed figures have now been made available at Open Science Framework (http://doi.org/10.17605/OSF.IO/7YB2M) along with code files to ensure that all figures can be reproduced. Reproducibility has been verified via internal

testing. Given the points about Github depositions being easily subject to change or deletion, we have removed this reference from the manuscript and now rely on the DOI'd link to http://doi.org/10.17605/OSF.IO/7YB2M instead.

The only exception among the figures listed is Figure S6, which represents a supplementary abstraction of village locations for the study population; this figure is produced using the latitude and longitude of villages in this study, and so has not been included. Given that this figure is minor and meant to help give some intuitive understanding to readers, we don't feel that omission of the underlying data jeopardizes reproducibility of the broader manuscript.

e) Please cite the location of the data clearly in all relevant main and supplementary Figure legends, e.g. "The data underlying this Figure can be found in S1 Data" or "The data underlying this Figure can be found in https://doi.org/10.5281/zenodo.XXXXX"

This information has been added to each relevant figure.

As you address these items, please take this last chance to review your reference list to ensure that it is complete and correct. If you have cited papers that have been retracted, please include the rationale for doing so in the manuscript text, or remove these references and replace them with relevant current references. Any changes to the reference list should be mentioned in the cover letter that accompanies your revised manuscript.

We have reviewed the references and made changes throughout. To our knowledge these are complete and correct, and do not contain reference to retracted papers.

DATA POLICY:

You may be aware of the PLOS Data Policy, which requires that all data be made available without restriction: http://journals.plos.org/plosbiology/s/data-availability. For more information, please also see this editorial: http://dx.doi.org/10.1371/journal.pbio.1001797

Note that we do not require all raw data. Rather, we ask that all individual quantitative observations that underlie the data summarized in the figures and results of your paper be made available in one of the following forms:

1) Supplementary files (e.g., excel). Please ensure that all data files are uploaded as 'Supporting Information' and are invariably referred to (in the manuscript, figure legends, and the Description field when uploading your files) using the following format verbatim: S1 Data, S2 Data, etc. Multiple panels of a single or even several figures can be included as multiple sheets in one excel

file that is saved using exactly the following convention: S1_Data.xlsx (using an underscore).

2) Deposition in a publicly available repository. Please also provide the accession code or a reviewer link so that we may view your data before publication.

Regardless of the method selected, please ensure that you provide the individual numerical values that underlie the summary data displayed in the following figure panels as they are essential for readers to assess your analysis and to reproduce it: Figs 2AB, 3, 4ABCDEFGHIJKLMNOP, 5ABCDEF, 6ABCDEF, 7, S1, S4, S5, S6, S7. NOTE: the numerical data provided should include all replicates AND the way in which the plotted mean and errors were derived (it should not present only the mean/average values).

IMPORTANT: Please also ensure that figure legends in your manuscript include information on where the underlying data can be found, and ensure your supplemental data file/s has a legend.

Please ensure that your Data Statement in the submission system accurately describes where your data can be found.

REVIEWERS' COMMENTS:

Reviewer #1

This paper discusses the spread of COVID-19 and its severity in indigenous populations. The author considers an individual-based network stochastic model instead of a traditional compartmental model as a novel approach as these indigenous communities are relatively small in population size and isolated from urban centers. Overall, the paper is well written, and the results section provides community information and individual-level COVID-19 transmission, mobility, and an excellent comparison of the effects of human behavioral changes on infectious numbers.

We appreciate the positive comments and attention to detail by the reviewer!

Comments:
1. There is a lack of information about the two models (Binomial GLMM, Multilevel M-splines hazard model in Table 1) used in the Results: outbreak section. I would suggest briefly describing why you choose the two models in the result section.

The two types of models (binomial generalized linear mixed model and multilevel hazard model) were included because we were interested in exploring two separate outcomes related to infection during simulations. The binomial GLMM allows us to assess differences in the probability of an individual *ever* (over the full course of simulation) being infected as a function of predictor variables, adjusting for repeated simulation runs. On the other

hand, the multilevel hazards model allows us to assess the time to event (i.e. earlier infection times). As such, the latter makes it possible to generate survival curves such as those shown in Figure 5.

We appreciate the reviewer calling attention to the need to describe this in the text. On page 37, we now include the following:

"Finally, we used Bayesian multilevel hazard models to characterize individual probabilities of infection over time. To do so, we modeled a flexible baseline hazard function using M-splines in addition to fixed (individual and community predictors) and random (community intercept) effects using the "stan_surv" function in the *rstanarm* package. Whereas the GLMMs described above assess the probability of an individual node ever being infected over the course of simulation as a function of predictor variables, the parametric hazard models assess time to infection event outcomes, and are used to generate the survival curves in Figure 5."

2. The animation Fig. S2 runs only until t=5-6, but in the paper, you talk about the disease dynamics at t=17-18. Please fix this (in the Total outbreak size - Community level Section).

Thank you for catching this error, the animation has been fixed and is no longer cut off prematurely.

3. It is confusing that the dependent variables in Table 2 are referred to as models throughout the paper (e.g.: Table 2: Model 3, Fig. 4i-l). Replace "model" with "column" or "variable".

Each column in Table 2 refers to a separate regression model, distinguished by the use of different dependent variables. However, given that this appears to have caused confusion, we have changed this to "column" throughout.

4. Please use one format only (uppercase or lowercase, but not both) when naming the figures and when citing them in the context. Right now, the figures are in uppercase, and they are referred to in paragraphs with lowercase letters.

We are happy to make this change, and have changed everything to capital letters for subplots (the words "Figure" and "Fig." already appear to be capitalized throughout).

5. Please cite this information "mean probabilities of intervillage travel and travel to town = 9% and 4% of days, respectively" (in Discussion Section paragraph 6).

The values reported here are from original empirical data that are already described in our methods section. To make it clear that this is the case, and that these values are not coming from another paper that should be cited, we have added explicit mention of this: "mean empirical probabilities of intervillage travel and travel to town are 9% and 4% of days, respectively".

6. I suggest changing the method of transportation: "boat motors" to "motorboats".

This change has been made.

7. I believe the TERGM framework in the Empirical data section is same as the STERGM framework discussed in the results section, if it is not, please mention it clearly.

These frameworks are indeed the same, and the text has been updated for consistency.

8. More explanation is required for the four sub-figures in animation Fig. S3, I do not see much difference among them. In addition to that, it would be helpful lowering the play speed of the animation and provide a pause/play button(s) with the animation.

The sub-figures for Fig. S3 merely represent 4 randomly selected independent model runs, and thus it is not surprising that major differences are difficult to detect (as this demonstrates how similar patterns can emerge repeatedly). To avoid confusion, however, we have modified the figure to show just one simulation run, because there was little information gained by having the multiple panels. Regarding the other points, these are great suggestions for improvement and we have slowed the play speed of the animation. Unfortunately, it is not straightforward to add a play/pause button to animated gifs, and it is not clear to us how to do this. With the slowed speed, however, we feel that this feature is not critical.

9. Please fix the link for the data and code (https://github.com/ThomasKraft/tsimane_epidemic), which is not working at the moment.

The link above was not working because this is currently a private repository. As per the directions of the editor, data/code are being made available on a more permanent archive (OSF) instead which is now linked in the paper. References to the git repository have been removed.

Reviewer #2

In this paper, the authors simulated the SEIRD transmission model on a stochastic social network of Indigenous communities in Amazon. The network has 7269 nodes and is parameterized with high-resolution empirical data on population structure, mobility, and contact networks.

This is a well-written paper. The results are interesting and consistent with the simuation results. The methods employed are explained in a clear and concise manner, making them easily comprehensible for readers. The flow of the paper is good, I believe the readers can effortlessly navigate through its contents. Personally, I found reading the paper to be a pleasant experience.

Upon careful examination, I couldn't identify any significant flaws in the paper. However, I did come across a few minor issues after conducting thorough checks.

I recommend the publication of this paper in PLOS Biology if the suggestions are considered.

We are glad the reviewer enjoyed reading the paper!

1. Page 30. The authors should explain why 'a truncated normal distribution with a theoretical maximum of 0.05 (5% probability of being infected after one day spent in town)' is being used. Is the infection risk follow normal distribution? Why is it a normal distribution?

The truncated normal distribution with maximum of 0.05 for infections seeded in town is an assumption of the model. This assumption is explained with the following text in the manuscript:

"At the outset of our model, all individuals are susceptible, and travel to a nearby market town is the only source of potential infection. The infection risk profile when travelling to town is specified as a truncated normal distribution with a theoretical maximum of 0.05 (5% probability of being infected after one day spent in town) with start and end at days 0 and 60, respectively (Fig. S4). This town infection profile is set to reflect the fact that nearby towns likely experienced a wave of infection spanning ~2 months (reducing town infection to 0 after this period ensures that the full dynamics of the epidemic play out without continuous seeding)."

Further, as stated in Table 1 under "Town infection risk", the maximum was set to correspond to the effective contact rate designated within-community, which is justified by multiple citations under "Transmissibility" in Table 1.

As for the normal distribution, we chose this profile because it approximates the distribution of confirmed COVID-19 cases during the initial outbreak period in Bolivia in summer 2020 (https://covid19.who.int/region/amro/country/bo). More fine-grained estimates from the local market town (San Borja) or region (Beni Department) are unavailable. This was not explained previously in the text, and so we have now added the following text (and appreciate the suggestion to do so):

"*A normal distribution was chosen because it approximates the empirical distribution of active COVID-19 cases reported for the country of Bolivia during the initial outbreak in summer 2020 (https://covid19.who.int/region/amro/country/bo)."*

Finally, as explained in the text above, a truncated distribution was used to ensure that town infections dropped to zero after a controlled amount of time, allowing us as analysts to observe the effects of endogenous spread without cases being continuously seeded from outside the population.

2. Page 34. In the second formula, age[10-26] is missing.

The current formulation is correct. Because age is represented as a categorical variable in this model, "Age[10-26]" is missing because it represents the baseline or intercept value to which other categories are estimated in comparison (and is thus not specified explicitly).

3. Page 48. Figure 4. A typo 'loess' in the last sentence.

'loess' was not a typo but is an acronym (for "locally estimated scatterplot smoothing") that was not properly defined as such in the text. This has now been fixed.

4. Page 56. The Figure S2 in the listed website doesn't match the description in paper.

Thank you for catching this. As noted in response to the other reviewer, this has now been fixed.
